# Comparison of the Primary Stability of Porous Tantalum and Titanium Acetabular Revision Constructs

**DOI:** 10.3390/ma13071783

**Published:** 2020-04-10

**Authors:** Nicholas A. Beckmann, Rudi G. Bitsch, Mareike Schonhoff, Klaus-Arno Siebenrock, Martin Schwarze, Sebastian Jaeger

**Affiliations:** 1 Clinic for Orthopedics and Trauma Surgery, Heidelberg University Hospital, 69118 Heidelberg, Germany; martin.schwarze@med.uni-heidelberg.de; 2 Department of Orthopaedic Surgery and Traumatology, Inselspital, Bern University Hospital, 3010 Bern, Switzerland; klaus.siebenrock@insel.ch; 3 National Joint Center, ATOS Clinics, 69115 Heidelberg, Germany; rudi.bitsch@atos.de; 4 Laboratory of Biomechanics and Implant Research, Clinic for Orthopedics and Trauma Surgery, Heidelberg University Hospital, 69118 Heidelberg, Germany; mareike.schonhoff@med.uni-heidelberg.de (M.S.); sebastian.jaeger@med.uni-heidelberg.de (S.J.)

**Keywords:** porous implants, tantalum, titanium, acetabulum, hip arthroplasty, hip replacement, revision hip arthroplasty, acetabular revision, primary stability

## Abstract

Adequate primary stability of the acetabular revision construct is necessary for long-term implant survival. The difference in primary stability between tantalum and titanium components is unclear. Six composite hemipelvises with an acetabular defect were implanted with a tantalum augment and cup, using cement fixation between cup and augment. Relative motion was measured at cup/bone, cup/augment and bone/augment interfaces at three load levels; the results were compared to the relative motion measured at the same interfaces of a titanium cup/augment construct of identical dimensions, also implanted into composite bone. The implants showed little relative motion at all load levels between the augment and cup. At the bone/augment and bone/cup interfaces the titanium implants showed less relative motion than tantalum at 30% load (*p* < 0.001), but more relative motion at 50% (*p* = n.s.) and 100% (*p* < 0001) load. The load did not have a significant effect at the augment/cup interface (*p* = 0.086); it did have a significant effect on relative motion of both implant materials at bone/cup and bone/augment interfaces (*p* < 0.001). All interfaces of both constructs displayed relative motion that should permit osseointegration. Tantalum, however, may provide a greater degree of primary stability at higher loads than titanium. The clinical implication is yet to be seen

## 1. Introduction

Total hip arthroplasty (THA) is a highly successful surgical intervention that is being performed with increasing frequency in cases of advanced osteoarthritis, and in patients of decreasing age [1]. The increased incidence of primary THA is accompanied by a corresponding increase in revision THA with the associated concerns of diminished bone quality, bone loss and compromised soft tissue [2]. Earlier interventions that addressed these concerns included the use of large structural allografts that had mixed results with loosening and migration rates of up to 70% [3]. Utilization of metal cages for large defects reduced the loosening rate to 14% at 6 year follow up [3,4]. In addition, the recognition of cement disease as a major cause of loosening and later failure in cemented constructs [5] led to the increasing use of cementless porous metal components that allowed for bone ingrowth that facilitated stable fixation. The introduction of porous metal implants with a range of accessory porous metal augments, buttresses and shims has led to a further improvement in revision THA outcome.

Currently, the most frequently used porous metal implants have either a tantalum or titanium porous metal surface and with press-fit implantation they provide a stable mechanical surface between implant and host bone in the short term (primary stability), and facilitate osseointegration in the mid and long term [6,7]. Tantalum in the form of Trabecular Metal™ (TM) (Zimmer Biomet, Warsaw, Indiana) is currently one of the more frequently used porous implants [8], and has been used to treat very extensive acetabular defects [9] as well as for neoplastic periacetabular lesions [10].

Optimal primary stability and ultimately adequate osseointegration and successful outcome is dependent on minimal relative motion at the component/bone interface. Prior experimental studies have shown that successful osseointegration occurs with relative motion between surfaces of up to 40µm, and that fibrous attachment occurs at 150 µm [11]. In addition, increased relative motion between components and bone can lead to particle generation and shedding that promotes later loosening and failure [12]. The use of additional components such as augments and buttresses increases the number of opposed surfaces and also the potential for increased relative motion in the construct as a whole, with possible consequences for the stability of the construct [13].

The aim of this study was to utilize an experimental biomechanical set up to evaluate the primary stability of a tantalum acetabular cup and augment construct as used in the treatment of larger acetabular defects [14], and compare the results with those of a similar porous titanium construct and augment that were published previously in an identical experimental set up [13].

## 2. Materials and Methods

**Figure 1:**
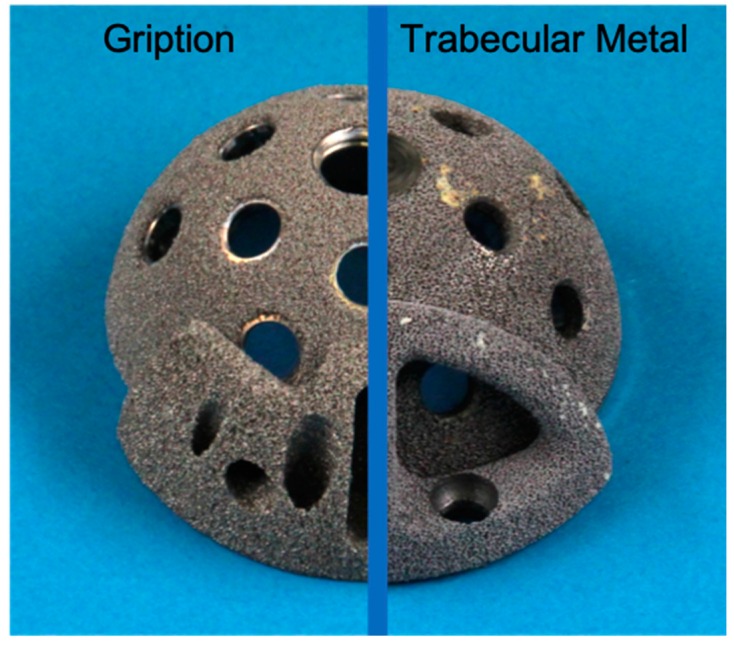
Photograph of titanium Gription cup and augment (**left**) and tantalum Trabecular Metal augment and cup (**right**) after implant explantation, demontrating the differences in their hole positions and augment geometry.

Six Trabecular Metal™ acetabular cup components of 56 mm diameter and corresponding Trabecular Metal™ augments of 54/56 × 1 cm size were utilized in our biomechanical set up (see Figure 1). We also utilized six large fourth generation composite left hemipelvises (#3405 Sawbones; Sawbones Europe AB, Malmo, Sweden) each with a created Paprosky 2b defect of 1 cm thickness that was segmental and constituted less than one third of the acetabular circumference. Each defect was created in an identical standardized manner at the postero-cranial aspect of each acetabulum, with the edge of the defect adjacent to the antero-inferior iliac spine. To accomplish this, the periphery of the defect was first marked on each hemipelvis and the central synthetic bone was reamed and burred to the peripheral mark and to 1cm depth. This created defect was then completely covered with a TM augment according to manufacturer’s instructions, and fixed to host bone with two 5.5 × 30 mm screws. Prior to acetabular cup implantation, premixed cement was then applied to the aspect of the augment that apposed the acetabular cup. A medium viscosity bone cement (Palacos R + G pro; Heraeus Medical Gmbh, Wehrheim, Germany) was used at this interface. The cement was vacuum mixed (Optivac Cement Mixing System; Zimmer Biomet, Warsaw, Indiana) and applied 120 s after the start of mixing. The cement was dispensed with a cement gun and 1.5 cm^3^ was hand-modeled on the augment surface. Excess cement was carefully removed from the multi-hole acetabular component. All cementing was done under standardized conditions with the same mean room temperature and humidity as in the prior experiment [13]. The acetabular cup component was then implanted according to manufacturer’s directions, and attached to host composite bone with one each of 6.5 × 40 mm and 6.5 × 30 mm screws, with the screws directed towards the sacro-iliac joint. The acetabular cup was press-fit as well as the rim defect would allow. All augments and cups were implanted by a single experienced surgeon (R.G.B.). 

Following implantation, the hemipelvises were secured along the sacral side of the ilium using polyurethane foam (RenCast FC 53 A/B; Goessl + Pfaff Gmbh, Karlskron, Germany) in a containment device [13]. The symphysis was also secured to a two-component casting resin block that had an attached stainless-steel ball on the under-side that was placed on a metal plate. This constituted a two-point pelvic fixation, with the pelvis fixed in only one degree of freedom to allow for multi-planar movement and rotation of the symphysis that mimics a physiologic fixation, as described in prior studies [13].

Optical markers of 0.8 mm diameter (uncoded passive white markers, GOM Item Number 21874; GOM Gmbh, Braunschweig, Germany) were placed in adjacent rows along the rims of the acetabular cup component, adjacent augment and the host bone [13]. These adjacent rows of markers were detected in grey-scale by a stereo camera system that provided 3D discrimination and recording of relative motion between components and bone during loading. This was achieved by using 3D point triangulation to calculate the 3D marker position in the x, y and z axes of the defined coordinate system [15]. The 3D relative motion in the x, y and z axes were measured simultaneously between the acetabular component and bone, acetabular component and augment and augment and bone using an optical measuring system (PONTOS, GOM Gmbh, Braunschweig, Germany). 

We pre-tested the hemipelvis set-up using a materials testing machine (MTS Mini Bionix 359; MTS Systems Corporation, Eden Prairie, Minnesota), with the load applied in the direction of the greatest load that occurs during the normal gait cycle, as defined by Bergmann et al. [16]. The maximum load during normal walking was found to be 233% of the individual’s body weight at 31 degrees of rotation around the x axis and 5 degrees around the z axis relative to the acetabular component system described by Bergmann et al. [17]. We arbitrarily chose a body weight of 80 kg for each specimen, as in our prior study [13], that was equivalent to 1.8 kN at 100% load at the hip during normal gait.

**Figure 2:**
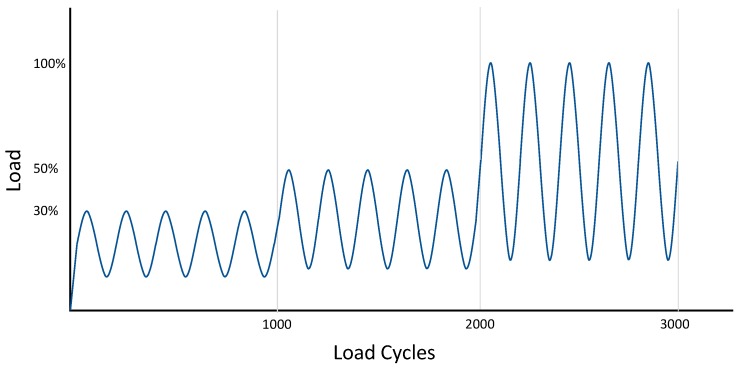
Schematic graph displaying the load applied for each sample over the 3000 test cycles.

Three load levels were chosen; 3–30% load (equal to 0.5 kN), 5–50% load (equal to 0.9 kN) and 10–100% load (equal to 1.8 kN) (see Figure 2). A total of 1000 cycles were applied sequentially in a sinusoidal wave-form at 1 Hz at each of the three load levels. To ensure good force closure between force plate and testing sample, 0.2 kN was applied prior to testing. The dependent variable (measured in µm, with average and variance) was the relative motion between components and bone, measured at the following groups of cycles; 1 to 50, 51 to 200, 201–500, 501–800, 801–995.

The results of measurements obtained as described above were compared to results obtained in an identical manner during a prior experimental set-up using Gription^®^ titanium components instead of tantalum components of the same diameter/size [13]. 

## 3. Statistical Analysis

Statistical evaluation was carried out descriptively (arithmetic mean, standard deviation, minimum and maximum). After confirmation of normal distribution using a Shapiro–Wilk test, a t-test of independent variables was performed. To evaluate differences in both groups during the cyclic loading, we performed an analysis of variance with repeated measures (ANOVA). The effects with regard to implant type and time points were evaluated. A *p*-value of ≤0.05 was considered significant. Results were presented using statistical graphics when necessary. Statistical evaluation was performed using Microsoft Excel (Microsoft Corporation, Santa Rosa, CA, USA), and the analytical software SPSS 25 (IBM Inc., Armonk, New York, USA). 

## 4. Results

One of the six samples was excluded, since the fixation of the hemipelvis in the containment device failed. Table 1 shows the average relative motion between the tantalum augment/cup, tantalum augment/bone and tantalum cup/bone interfaces at 30%, 50% and 100% load for the remaining samples and compares it to the average relative motion between titanium cups/augment, titanium augment/bone and titanium cup/bone interfaces.

**Figure 3:**
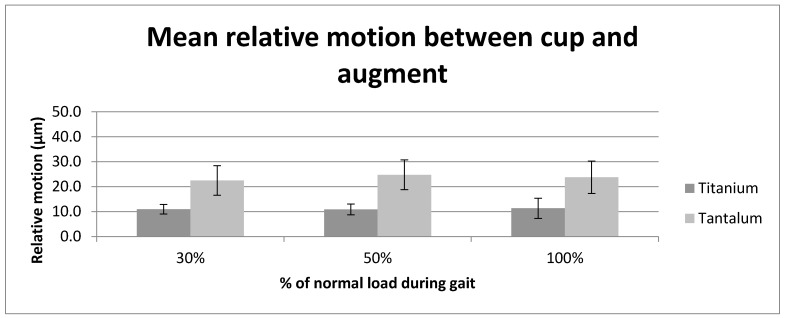
Graph displaying the average relative motion (µm) at the tantalum and titanium augment and cup interfaces at the three tested load levels (30%, 50% and 100% load).

The t-test revealed a statistically significant difference in the relative motion between titanium augment/cup and tantalum augment/cup at all load levels (30% load: t(8) = −20.34, *p* < 0.001; 50% load: t(8) = −30.06, *p* < 0.001; 100% load: t(8) = −14.32, *p* < 0.001) (see Figure 3). 

**Figure 4:**
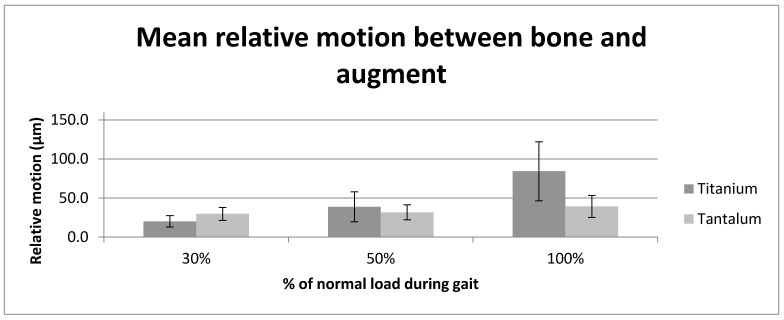
Graph shows the average relative motion (µm) between the tantalum and titanium augment and adjacent composite bone at the three tested load levels (30%, 50% and 100% load).

The titanium augment/sawbone interface displayed less relative motion at 30% load than the tantalum augment/sawbone interface (30% load: t(8) = −8.81, *p* < 0.001). At 50% (t(8) = 1.59, *p* = 0.151) and 100% (t(8) = 15.47, *p* < 0.001) load there was an increased average relative motion of the titanium augment/Sawbone interface when compared to the relative motion at the tantalum/Sawbone interface (see Figure 4). 

**Figure 5:**
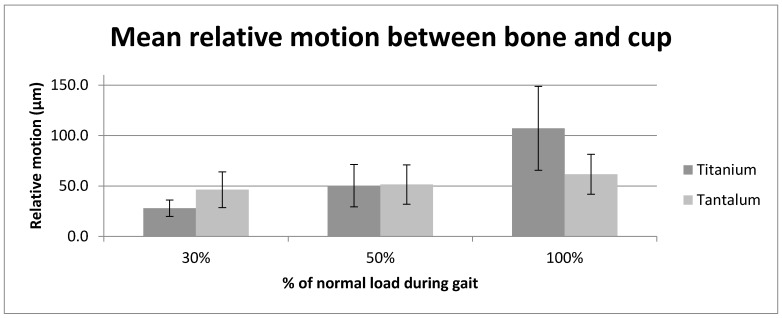
Graph showing the average relative motion (µm) between the tantalum and titanium cup and composite bone at the three tested load levels (30%, 50% and 100% load).

At 30% load, the titanium displayed significantly lower relative motion (t(8) = −13.00, *p* < 0.001) at the bone/cup interface, while at 50% load (t(8) = −0.20, *p* = 0.843) and at 100% load (t(8) = 11.76, *p* < 0.001) the tantalum displayed lower relative motion (see Figure 5).

No significant difference was noted at the augment/cup interface with regard to the load level (F(2, 16) = 2.87, *p* = 0.086). The load level did, however, have a significant effect on the relative motion at the bone/augment (F(2, 16) = 352.66, *p* < 0.001) and bone/cup (F(2, 16) = 331.96, *p* < 0.001) interfaces. 

## 5. Discussion

The incidence of revision total hip arthroplasty (RTHA) is continuing to increase, particularly in younger patients [1,18] and is predicted to increase to 14.5% of all THAs and to increase by 174% from 2005 to 2030 [1].

Aseptic loosening has been reported to be the major reason for THA revision [2,18], and the frequently associated osteolytic defects that result from particulate debris and component wear can present a significant surgical challenge [2,19,20]. Revision THA consequently has a greater incidence of failure than primary THA because of the compromised soft tissue, bone loss and increased complexity of the procedure. This has prompted the ongoing search for improved components. Cemented acetabular cups allow only bone ongrowth rather than bone ingrowth [21,22] and have been associated with poor integration into the sclerotic host bone, increased rates of bone resorption and increased difficulty with later revision procedures [23]. Porous coated uncemented acetabular implants depend upon press-fit implantation to provide adequate primary stability during the surgical and early postoperative phase and secondary stability from later adequate osseointegration [22].

Currently tantalum and titanium are the most frequently used metals in uncemented porous components due to their biologically inert nature and their physical properties that are close to those of cancellous bone [24,25,26,27]. In addition, a recent study by Brüggemann et al. has shown little systemic response to tantalum implants, underscoring their safety in joint replacement procedures [28]. A large body of literature documents the success of porous trabecular tantalum constructs in RTHA [4,29,30]. In contrast, there is a relatively small body of literature documenting outcome with titanium constructs that vary in type and physical properties as a result of differing manufacturing processes [6,30].

The goal of our study was the evaluation of relative motion occurring at all interfaces of an implanted TM acetabular/augment construct and comparison of the results with the previously recorded relative motion occurring at the same interfaces of a porous titanium (Gription^®^) acetabular cup/augment construct, implanted under identical technical and environmental conditions. The inclusion of an augment in the construct added an additional interface (cup/augment) with the potential for additional relative motion. In all our tantalum and titanium constructs we found minimal relative motion at the cup/augment interface at all load levels, and we therefore interpreted this interface as having no significant negative impact on the stability of the construct as a whole. The tantalum and titanium constructs also displayed minimal relative motion of 30–50 µm at the bone/cup and bone/augment interfaces at the 30% and 50% load level. At the 100% load only, the Gription constructs displayed increased relative motion of the bone/cup and bone/augment interfaces of 107 and 84 µm, respectively. This may be due to the properties of the materials and implants, and their respective elastic modulus. Differences in the coefficient of friction alone have been shown in a prior study to have little impact on the primary stability of the acetabular component [31]. In all instances these values are below the previously recorded levels of relative motion that are thought to result in fibrous attachment. Prior in-vivo animal studies and studies on human autopsy bones have shown that successful osseointegration occurs with up to 40 µm relative motion between implant and bone, and fibrous attachment occurs with 150 µm relative motion [11,32]. It has also been shown that successful osseointegration can occur with bony attachment that involves substantially less than 100% of the bone/implant interface [33,34] and most of the osseointegration occurs around the acetabular rim, and decreases towards the pole [34,35]. One study by Bondarenko et al. showed that osteoporotic bone has worse osseointegration than healthy bone, and also that the implant can have a significant effect on the osseointegration, or bone-implant-contact [36]. In their study the tantalum implant Trabecular Metal^®^ and the Trabecular Titanium^®^ showed better osseointegration than the titanium implants Stiktite^®^, titanium with Gription^®^ coating or Tritanium^®^ [36].

The minimal levels of relative motion between porous implant and bone promote successful osseointegration, secondary stability and good surgical outcome, as documented in clinical reports. In RTHA in particular, tantalum components have been reported to have excellent results in complex cases, even with large bone deficiencies [21,37,38]. The ancillary use of porous tantalum augments as buttresses in cases of insufficient acetabular rim support has also been reported to have superior results [2,39,40]. Konan et al. reported a 96% survivorship of the TM acetabular component and good functional outcome at a mean 11 year follow up in patients with Paprosky 11 and 111 defects [41]. Morselized allograft was used in most cases, and no augments. Survivorships of 10 years for tantalum cup/augment constructs have been reported by a number of authors as 91–97% [20,27,37]. There are very few studies of porous titanium components in RTHA, and also little research of the titanium Gription cup/augment constructs in RTHA. One study evaluating 146 Pinnacle Gription cups, 1 of which was used in combination with an augment, showed good short-term results after RTHA [42]. In addition, studies with Gription augments used with various other cup types have shown good functional results [2]. However, there have been recent reports of studies that used other titanium components of different composition and manufacture, such as Trabecular Titanium and Tritanium^®^ [43]. Hosny in 2018 reported a 98.4% aseptic acetabular cup survivorship at mean follow up of 87.6 months using Tritanium^®^ revision cups in 62 patients with Paprosky 1–3 defects [6]. No augments were used. Delanois reported a 97% aseptic acetabular cup survivorship in 35 patients with a mean 6 year follow up, also using Tritanium^®^ cups [23]. 

Our study has several limitations. Although the tantalum and titanium set-ups were done under identical technical and environmental conditions with implantations performed by a single surgeon in all cases (RGB), small differences in implantation technique cannot be ruled out.

Results for the Gription^®^ samples cannot be extrapolated to other types of titanium implants in biomechanical experiments or in the clinical scenario. There are currently several different titanium product lines that differ in composition, architecture and manufacture, with differing biophysical properties. 

We chose to use synthetic composite bone (Sawbone^®^) rather than cadaveric bone because of the uniformity in composition that is particularly important when working with a small sample, although the biomechanical properties are not identical to bone. Our results therefore may not reflect the clinical scenario. 

Increasing loads were applied up to 100% of average normal body weight (80 kg), that was our best estimate of the limited weight bearing experienced during the postoperative period. The maximum load experienced during normal walking conditions is 233% of body weight [16,17]. Joint loading was applied only in the direction of maximal load as defined by Bergmann, and did not reflect the cyclical pattern of loading during normal walking conditions. 

## 6. Conclusions

The samples in our study showed minimal relative motion that should promote successful osseointegration. The Gription construct showed more relative motion than TM at the cup/bone and augment/bone interfaces at 100% load only, that was below the value thought to promote fibrous attachment. Relative motion at the cup/augment interface of both TM and Gription constructs was of a degree that should not negatively impact the stability of the construct as a whole. Our biomechanical results are consistent with the positive clinical experience with TM components. There are too few reports on Gription constructs to make any clinical correlation, but our test results suggest that they should function satisfactorily.

## Figures and Tables

**Table 1:** Table showing the mean and standard deviation (SD) of the relative motion (µm) of tantalum (Trabecular Metal) and titanium (Gription) implants at the respective implant/bone interfaces and load levels.

Interface	Augment/Cup		Bone/Augment		Bone/Cup	
Implant Material	Titanium	Tantalum	Titanium	Tantalum	Titanium	Tantalum
Load	Mean (SD)	Mean (SD)	Mean (SD)	Mean (SD)	Mean (SD)	Mean (SD)
30%	11.0 (1.9)	22.5 (6.1)	20.0 (7.3)	29.7 (8.1)	27.9 (8.0)	46.3 (18.6)
50%	10.9 (2.1)	24.7 (5.7)	38.7 (17.8)	31.7 (9.7)	50.2 (18.6)	51.4 (19.8)
100%	11.3 (4.2)	23.7 (6.6)	84.3 (40.2)	39.4 (15.0)	107.2 (44.0)	61.6 (20.5)
